# On the Use of Glycerine in Diseases of the Ear

**Published:** 1849-09

**Authors:** Thomas H. Wakley

**Affiliations:** Surgeon to the Royal Free Hospital, London


### SURGERY.

*On the use of Glycerine in Diseases of the Ear.* By Thomas H. Wakley, Esq., Surgeon to the Royal Free Hospital, London.—The anxiety which is always manifested by the public to take advantage of any new discovery or successful mode of treatment connected with the removal of disease, affords a strong proof of the confidence which is generally entertained by the community in the power of medicine as it is practised in this country. In this respect, a marked difference is shown in the conduct of the professional and non-professional portion of society. This is not extraordinary, as afflicted persons naturally seek for the readiest mode of obtaining relief. The medical practitioner, on the contrary, first considers the rationality of any proposed method of effecting a cure. His studies have taught him to reflect and to reason, and the results of experience have admonished him not to form conclusions too hastily, or to generalize too widely from restricted data. But it must not be inferred, that because the medical practitioner is less prompt than the public in resorting to the aid of a new agent, that his confidence in the powers of the healing art is less forcible, or is less deeply fixed in the foundations of his judgment, than is that of the non-professional community; on the contrary, the confidence which he feels is not the offspring of an unreflecting, ill-sustained faith—it is not a weak superstructure, raised upon the narrow basis of a single fact, but arises from, and is sustained by, the broad and solid foundations of science, reason, and experience. It is well for society and the progress of knowledge that the minds of medical practitioners are thus trained and disciplined; for if it were a custom with them, without hesitation and reflection, to adopt and sanction every newly-announced successful plan of treating disease, the subsequent failures, in a great majority of the novel plans, would soon destroy all confidence in the practice of medicine, not only in the public mind, but amongst physicians and surgeons themselves. Probably one of the causes which has tended in a great degree to establish and sustain the confidence of the community in the practice of medicine, has been the judicious caution which practitioners of eminence have exercised before they have resorted to new means of cure; and secondly, the candor and integrity which they have so often displayed in publishing the results of their experiments and experience. It is honorably felt that much deliberation and caution is due alike to a noble science, to the just claims of society, and to the exalted character of a dignified profession. If every example of the successful treatment of disease, by a new remedy, were to be published, the minds of practitioners, if not strongly fortified by previous study, and habits of thoughtful investigation, would become in danger of being involved in confusion by the dazzling announcements of numberless triumphant experimentalists. It is, therefore, forcibly felt by the profession, that before a new mode of treating disease be recommended, something more substantial than speculation or hypothesis should be available to warrant its adoption. Reflections of this kind have induced me to pause for a very considerable period before I determined respectfully to submit to the consideration of the profession, the humble pretensions which I am desirous of establishing for *glycerine* in the treatment of certain forms of deafness. The strong conviction which I entertain as to the utility and value of this remedy is the only apology I can offer for claiming, even for a moment, the attention of the profession on such a subject. I have no new doctrine to inculcate; no new discovery in physiology to enforce; no "great fact" in pathology to disclose. The sum total of my aim on this occasion is, to contribute a fact to our therapeutic store, which, although it may be regarded by some as a very insignificant item, is one which I think ought to be very generally known. I have already adverted to the confidence felt by the public in the capabilities of medicine, as a practical science, and to the eagerness shown by society to take advantage of any newly-announced medical or surgical remedy. Unfortunately, that unsuspecting desire to seize, with blind faith and hope, on any new proposal for curing disease, is, in this country, the fertile source and support of all those partial, detached, and empirical systems of practice, which are, and ever must be, denounced and repudiated by every well-educated practitioner.

The medical officers connected with the public institutions of this vast metropolis command the most ample opportunities of observing the strong tendencies of the public feeling with respect to the adoption of new remedies. Scarcely is a new fact of any importance connected with the cure of disease published in the periodical journals, when the "out-patients" at the public hospitals make the proposed remedy the subject of common conversation amongst them. Frequently, the applicants for relief even ask to be treated on the "new plan," and sometimes they request to be given some of the "new medicine."

One of the most striking instances of the lively effect produced by the first announcement of a "new plan" of treatment, was afforded, in the summer of last year, by the publication, in The Lancet for July 1, 1848, of the first of a series of valuable papers by Mr. Yearsley, entitled, "On a New Mode of Treating Deafness." Immediately after the appearance of those papers, an influx of a new class of patients was observable in the Royal Free Hospital. The members of that new class of persons were afflicted with deafness, and often was the remark made by them, "I wish, if you please, to be treated upon the new plan;" or the question was asked, "if there had not been discovered a cure for deafness?" Such inquiries from patients suffering under actual disease, many of whom stated that they were deprived of the means of obtaining a livelihood, in consequence of their infirmities, suggested the questions—"What ought the surgeons of a public general hospital to do, under such circumstances?" "Ought these patients to be rejected at this place, and transferred to the institutions specially devoted to diseases of the ear?" It appeared to be just that the patients should be received.

Accordingly, I resolved to attempt to confer a benefit on the applicants, by adopting and following out the plan of treatment recommended by Mr. Yearsley; and this resolution was carried into practice with results which rapidly increased the number of expectant patients. Would it have been right, I ask, to refer such patients to other institutions by rejecting their supplications for relief, or, worse still, to consign them possibly to a class of not over-scrupulous practitioners, who profess to work miracles, but fortunately whom medicine does not claim, or in any manner recognise, as her legitimate children. Let it not be considered, from these remarks, that I deprecate a division of labor in the practice of medicine and surgery. On the contrary, I distinctly acknowledge, that an intense scientific application devoted to "specialties" in the investigation and treatment of disease has been productive of highly useful and important results. At the same time, such divisions of labor cannot, in my opinion, justify the surgeon of a public hospital in neglecting the consideration and treatment of any class of diseases which properly fall within the field of his observation. I need not dwell upon the importance of the sense of hearing. Why should a surgeon admit that a disease of the eye or the tongue properly demands his interference and best attention, and then altogether reject as unworthy of notice or consideration the deranged structure of the organ of hearing. The principles of diseased action are the same in all the vital organs, although peculiarities of tissue produce different morbid results.

During the existence of the first flush of success, the value of Mr. Yearsley's new method of treatment may have been over-estimated. This was not his fault; and the fact cannot be disputed, that Mr. Yearsley openly and candidly submitted his views to the notice of the profession. However, it happened that poor patients, in considerable numbers, claimed, at the portals of the public hospitals, to be recipients of the advantages of the new discovery, and I then thought, and still think, that it would have been ungracious and unfeeling to have rudely directed the sufferers to apply elsewhere for relief.

The adoption of the plan of treatment recommended by Mr. Yearsley was an appropriate recognition of the value of his labors. The success which attended the use of the simple operation which he recommended was, as I have already remarked, very striking in the first instance. In several cases, the effect of the application of the wetted cotton, in which the tympanum had been perforated by ulceration, was even extraordinary. But it too frequently happened, that the relief obtained was of an ephemeral duration. On applying the wetted cotton, the power of hearing, in several instances, which had been lost for a very long period, was instantaneously restored—an event which excited the most profound astonishment in the minds of patients and their friends. Too soon, however, was it perceived that the newly-acquired power gradually subsided, and the sense of hearing returned to its previous imperfect condition. The relapse frequently produced a feeling of dejection in the spirits and hopes of the patient which it was painful to witness. The benefit derived from the application, in the first instance, was undoubted, and could not be mistaken: hence arose the question—Why was it of so evanescent a character? This inquiry naturally suggested a minute investigation into the nature of the materials employed, and also their immediate and remote influence on the parts to which they were applied. A brief investigation, a few experiments, and an attentive consideration of the subject, induced me to attribute all the conferred advantages to the effects produced by the water, and to reject the cotton as nearly, if not entirely, useless. It even appeared that after the water had evaporated, the retained dry cotton became an additional impediment to the function of hearing. What was to be done? What were the indications which such facts seemed to establish? Evidently the use of some agent, which, by offering a successful resistance to evaporation, should retain its moisture, and continue to lubricate the auditory canal. Clearly enough, it was from the moisture that the benefit was obtained, and from a continuance of the moisture was the advantage to be prolonged. On duly considering all that I had observed, it appeared to me that *glycerine* was the only agent which was at all likely to accomplish the object I had in view. After consulting with Mr. Lloyd Bullock, of Conduit-street, relative to the composition and properties of glycerine, my opinion as to the propriety of giving it a trial was confirmed; and Mr. Bullock kindly manufactured for me a small quantity of the preparation in its purest form. This portion I obtained from Mr. Bullock in the first week of August, 1848, and employed it immediately in several cases, with apparently the most complete success. One of the patients, aged nineteen years, was a relative of Mr. Braithwaite, the celebrated engineer. In this instance the deafness had existed from infancy. Reports of four of these cases will be found amongst those attached to this paper. They were the first I treated with the new agent. In all these patients the wetted cotton had failed to produce a lasting benefit. Two of the four patients are now completely cured; and the other two are so far recovered as only to find it necessary to resort to the glycerine at distant intervals. The success of the new remedy, in these and many other instances, has attracted much notice; and I have now used the glycerine in upwards of three hundred cases of deafness. On many occasions it has been employed without any advantage whatever. In other instances the benefit was considerable for a short time, and then disappeared. In numerous cases, however, by the use of it, the power of hearing has been completely restored.

It was only after much experience in the application of glycerine, and from observing its action in a great number of cases, that it could be ascertained what were those conditions of the ear in which it was most likely to prove of advantage. Contrary to what might have been anticipated, the use of the remedy was successful in persons in whom the deafness had been of many years' duration—one, for example, thirty years, and also in cases where the existence of the malady could be traced to the eruptive fevers of childhood. In instances of deafness caused by inflammation, followed first by suppuration, and then by a horny, dry condition of the auditory canal, the application of glycerine has been attended with signal advantage. Equally marked and peculiar is the success when it is used in cases where there is a partial or total absence of ceruminous secretion. In many instances of deafness belonging to these classes of cases, the employment of glycerine has been followed by a perfect restoration of the power of hearing. In other examples of deafness, where the membrana tympani had evidently become thickened and hardened, and on examination with the speculum, denoted a whitish or pearly appearance, the use of the glycerine was followed by strikingly beneficial and gratifying effects. It is evident, therefore, that the application of glycerine is equally admissible, whether the tympanum be in a sound state, or whether it has been destroyed by ulceration.

A description of the composition and properties of glycerine, abridged from Turner's "Elements of Chemistry," may not be uninteresting on this occasion.

Glycerine was discovered by Scheele, and Chevreul proved its exact composition and constitution. Its formula is $C_6H_7O_5 + aq$. It is found in fatty oils combined with oleic, stearic, and margaric acids; its specific gravity is 1.252. Glycerine is a syrupy liquid, miscible both with alcohol and water, insoluble in ether, slightly inflammable, inodorous, and of a sweet taste.

The most convenient mode of preparing it is by the saponification of olive oil, by means of litharge and a little water. Sulphuric acid will separate the oily matters, leaving an aqueous solution containing the alkaline salt along with the glycerine. The mixture is evaporated to dryness, and treated with alcohol, which again dissolves the glycerine, and leaves the alkaline sulphate undissolved. The glycerine may be purified from oxide of lead, by passing through it a current of sulphuretted hydrogen.

### *Abstracts of Reports of Cases treated with Glycerine.*

Miss B——, aged nineteen; Lambeth; is healthy; has been deaf fifteen years. August 7th. This was a favorable case for the glycerine. Membrana tympani sound in both ears. The meatus externus of each ear is quite dry. A "singing noise" in both ears is continually present. Has been deaf since she had scarlet fever, fifteen years ago. The auditory passages are tender to the touch, and the lining membrane appears unusually white, and the meatus very small. The ears were washed and dried, and the external meatus well covered with the glycerine, which proved eminently successful. In a few minutes, to the great astonishment of her parent, she could hear a whisper. She applied again on the following Monday, and then stated, apparently with great joy, that she had been to church the day previous, and had heard the voice of the clergymen for the first time in her life. In this case the glycerine was applied three times a week for six weeks; after that period it was no longer needed. A cure had been effected, and the ceruminous secretion restored. No return of the deafness has occurred.

Miss H——, Coppice-row, Clerkenwell, aged twenty years, applied to me on the 7th of August. Has good health; states that at the age of twelve years she had measles, and has been deaf to a distressing degree ever since that time. A discharge flowed from both ears during the attack of measles; when it ceased, the deafness became extreme, and when I first saw her it had continued eight years. On examination, the lining membrane of both ears was found to be hard and dry. The tympanum of each ear was sound; there was no ceruminous secretion. She states, that after washing her ear she can frequently hear better for a short time; but so soon as the organs become dry the hearing is lost. In order to make her hear at all, the voice must be raised to a very high tone indeed. On the first application of the remedy, the improvement was very marked in both ears. In six weeks she discontinued her visits, having previously stated that she was perfectly cured. I saw this patient only a few days since; she still hears well.

C. A——, nineteen years of age. August 9. This patient was sent to me by Mr. Weedon Cooke. He is a scrofulous, unhealthy-looking lad. He says, that when he was five years old he had scarlet fever, and soon after a slight discharge flowed from his ears. His mother states that this discharge was highly offensive; at that time there was very slight deafness. This discharge continued, varying in quantity, for six months, when it ceased, and the patient then became deaf in both ears, and the deafness has continued ever since. On examination, the membrana tympani was found to be sound in each ear, but the lining membrane of the external meatus was much thickened, hard, and of a whitish appearance. Never remembers having had any "wax" in his ears. I stated to the medical gentlemen who were present, that I considered this to be a favorable case for the glycerine. Having prepared the aural canals, as in the other cases, the glycerine was applied, the power of hearing being extraordinarily increased. The glycerine was used many times, and always with the same successful result. When the ears are under the influence of glycerine, the patient can hear when he is addressed in an under tone of voice. But I am doubtful whether a complete cure will be effected in this case.

E. M——, Dean street. Aug. 15. Aged nine years, deaf and dumb. This child, the father said, had never been known to hear, and he believed if a pistol were to be fired near his ear, he would not take the slightest notice of it. The ears are very small. Upon examination with the speculum auris, the membrana tympani was found quite normal. The throat is well formed. The tonsils and uvula, in situation and size, are quite natural. The ears were well saturated with the glycerine. The hearing was then tested in the presence of Mr. Whidburn, Mr. Robinson, Mr. Moorhouse, and other medical gentlemen. His name was called loudly behind him ; he turned his head at each call ; we then directed him to signify with his fingers the number of times he was called by his father, who was situated at the other end ot the room. That the boy heard was apparent to all present, by the accuracy with which he answered by means of signals. I saw this child several times, but we could not improve at all upon the benefit he received from our first operation. His parents have, however, commenced teaching him words. I have not seen anything of the child for some time. It is certain that he received a certain amount of benefit from the glycerine.

Miss R——, Chad's-place, Gray's-inn-road ; aged eighteen years. August 15th, 1848. Is not enjoying good health ; has been deaf from infancy, in both ears. The malady followed an attack of small-pox. The lining membrane of both ears is pale, hard, and unyielding to the touch of an instrument. The tympanum of each ear appears perfect ; she never found any secretion in either ear ; had consulted several surgeons, who had applied blisters, essential oils, &c., but without the slightest benefit. She was sent to me by Mr. Jackson. The ears were carefully cleaned by means of cotton held within the blades of a pair of forceps, and dipped frequently in warm water. The canals were then rubbed with dry cotton, held in a like manner. Then the glycerine was applied by the same means, the cotton, well soaked in it, having been repeatedly passed backwards and forwards in each external meatus, care having been taken to apply it to the tympanum. The effect was particularly well-marked. This patient was under treatment for two months—a complete cure was effected. I heard of her a few days since, and her mother says she continues to hear perfectly.

E. P——. Aug. 16 Sent to me by Mr. Robinson, of Gower-street. Aged fourteen. Her mother cannot date her deafness to any cause ; states that she has gone through the routine of children's diseases ; does not recollect whether deafness followed closely upon any fever ; states that she is so deaf, that she is quite useless for domestic purposes, and that she had been under treatment by many surgeons, no benefit following. The ears were quite dry ; upon washing them, numbers of scales of the epidermis came away, and upon inquiry, she states that "scurf" frequently comes out of her ears ; she can always hear better after washing them. From these facts, it is quite evident that deafness was, to a certain degree, due to a deficiency of the ceruminous secretion. The glycerine was applied in the usual manner. Almost immediately, she heard the tick of a small Geneva watch quite distinctly. The glycerine was used during six weeks, and a complete cure was accomplished.

Miss J. M——, Devonshire-street, Mile-end. August 20. Has been deaf in both ears during the last five years. Cannot trace the deafness to any particular cause. There has not been any discharge. There is constantly an uneasy sensation in the ears. Membrana tympani imperforate. Not any secretion in either ear. On applying the glycerine, she heard a watch tick with both ears. In this case the patient cannot hear without using the glycerine. The condition of this patient remains stationary.

Mrs. D——, aged forty-eight, White Horse-lane, Stepney. September 3d. Is very healthy; sanguine temperament. States that for forty years she has been almost deaf in the right ear, from the effects of a blow given by her school-mistress. She can hear slightly in the left ear, but is constantly annoyed by a singing noise, and sometimes there is a dull pain over the mastoid process. She has not had fever. There has not been any discharge from the ears, except just after the time when the blow was inflicted, when there flowed a thin fluid from the right ear for about three months. When the discharge ceased, the deafness commenced, and has continued from that period. Upon examining the membrana tympani of the affected ear, no lesion in it could be discovered. The canal of the left ear was quite dry, and without any secretion. She states she can hear with both ears better in wet weather. Upon the application of the glycerine in the usual way, she heard the tick of a watch with both ears, but most distinctly with the right. Previously this had been the deaf ear. The glycerine has been repeated many times, and its use is still continued with the same success. This case was one of the most interesting that I have seen.

Mrs. P. O——, Adelaide-terrace, Islington, aged fifty-six years. September 3, 1848. Has been deaf thirty years; dates the malady from a very severe attack of rheumatism in the head. The meatus of each ear is exceedingly hard, and the membrana tympani much thickened, and of a pearly whiteness; not the least secretion. The membrana tympani cannot be touched without causing pain to the patient; many means of relief had been tried, but without effecting any improvement.

The auditory canals were carefully washed and dried, and then coated with glycerine by means of a camel-hair brush. An improvement in the state of the patient's hearing was very soon manifested. Her son, who accompanied her, said, that for years it was painful to converse with her. Knowing how great was her defect of hearing, she seldom attempted to provoke conversation.

When the glycerine had been introduced a few minutes, her son addressed her in an ordinary manner, and to his astonishment she heard him perfectly. In a few days afterwards she could hear carriages distinctly, and the noise made by the wheels passing over the stones was painfully unpleasant to the newly-excited organ. She also heard thunder distinctly, not having previously heard it for thirty years. The glycerine still acts effectually; but when her ears are not under its influence, she is as deaf as ever. The hearing continues distinct by applying the glycerine twice a week. If she removes the glycerine, and renders the auditory passages dry, the patient becomes quite deaf. The relief obtained while the ears are under the influence of glycerine is very great—an interesting fact, when it is recollected that the deafness of the patient had been of thirty years duration.

M. T——, Tottenham-court-road, has been deaf eight years. Sept. 4th, 1848. He cannot refer its existence to any precise cause. The aural canals are quite dry; the meatus is very large, and the membrana tympani is very easily seen, and found to be quite sound. This patient states that he is in the habit of moistening his ears frequently during the day, always hearing immediately after. From this statement, the success of the glycerine promised to be certain. Not having any glycerine by me at the time, I merely moistened the ears with water, when the hearing was immediately much improved. I then carefully dried both ears, and the power of hearing again relapsed to its former low degree. On the following morning the patient again attended, the glycerine was applied, and the effect produced was even greater than had been anticipated. The patient always receives the same amount of benefit from the glycerine, and the force of its action is as evident now as when it was first used. The beneficial effects produced by each application of the glycerine continue for six days. I do not believe that any cure will be effected in this case, as it is probable he will always be obliged to use the glycerine.

Mrs. S——, Greenwich, aged thirty-eight years. Dec. 15th. Considers her deafness to have been caused by a descent in a diving-bell ; she is deaf in both ears, which presented the same appearance in such cases. The aural canals are hard, polished, and dry ; she is so exceedingly deaf that the tone of the voice must be very considerably raised to make her understand. After the glycerine had been applied, she conversed with the friend who brought her in a tone of voice ordinarily used in common conversation. This was a very marked and successful case.—*London Lancet.*

